# Transpiration Response and Growth in Pearl Millet Parental Lines and Hybrids Bred for Contrasting Rainfall Environments

**DOI:** 10.3389/fpls.2017.01846

**Published:** 2017-10-30

**Authors:** Susan Medina, S. K. Gupta, Vincent Vadez

**Affiliations:** ^1^Crop Physiology Laboratory, International Crops Research Institute for the Semi-Arid Tropics, Patancheru, India; ^2^Integrative Crop Ecophysiology Group, Plant Physiology Section, Faculty of Biology, University of Barcelona, Barcelona, Spain; ^3^Crop Improvement Theme Asia Program, International Crops Research Institute for the Semi-Arid Tropics, Patancheru, India

**Keywords:** adaptation, environment, rainfall, pearl millet, VPD response, FTSW threshold, leaf development, growth

## Abstract

Under conditions of high vapor pressure deficit (VPD) and soil drying, restricting transpiration is an important avenue to gain efficiency in water use. The question we raise in this article is whether breeding for agro-ecological environments that differ for the rainfall have selected for traits that control plant water use. These are measured in pearl millet materials bred for zones varying in rainfall (8 combinations of parent and F_1_-hybrids, 18 F_1_-hybrids and then 40 F_1_-hybrids). In all cases, we found an agro-ecological variation in the slope of the transpiration response to increasing VPD, and parental line variation in the transpiration response to soil drying within hybrids/parent combinations. The hybrids adapted to lower rainfall had higher transpiration response curves than those from the highest rainfall zones, but showed no variation in how transpiration responded to soil drying. The genotypes bred for lower rainfall zones showed lower leaf area, dry matter, thicker leaves, root development, and exudation, than the ones bred for high rainfall zone when grown in the low VPD environment of the greenhouse, but there was no difference in their root length neither on the root/shoot index in these genotypes. By contrast, when grown under high VPD conditions outdoors, the lower rainfall hybrids had the highest leaf, tiller, and biomass development. Finally, under soil drying the genotypes from the lower rainfall accumulated less biomass than the ones from higher rainfall zone, and so did the parental lines compared to the hybrids. These differences in the transpiration response and growth clearly showed that breeding for different agro-ecological zones also bred for different genotype strategies in relation to traits related to plant water use.

**Highlights**:

• Variation in transpiration response reflected breeding for agro-ecological zones

• Different growth strategies depended on the environmental conditions

• Different ideotypes reflected rainfall levels in specific agro-ecological zones

## Introduction

Crops must enhance their productivity with less available water. The tolerance, or fitness, of a particular genotype to water limitations depends on its ability to match its water requirements to the water supply in specific environments (Vadez et al., 2013). Next to adapting the phenology and crop duration to fit water availability, genotypes with different canopy sizes are expected to have different water demands. Restricting water use by stomatal control is another avenue to fit water demand to water supply, although it may lead to either water being lost through evaporation or a lost opportunity for carbon fixation, suggesting that water saving is not a one-fit-all strategy. Therefore, understanding and analyzing traits that contribute to crop fitness to specific stress environment, especially those that control plant water use, is a prerequisite to breed adapted cultivars to specific environments. The hypothesis of this paper is that some of these traits could have been influenced by the breeding history.

Pearl Millet (*Pennisetum glaucum* L.) is a major crop in India and this country is the higher producer of this crop, this cereal is able to grow in the most arid zones. Its cultivation is being developed in the north arid and semi-arid regions of this country, these agro-ecological zones varying principally in the rainfall level. The *Lower rainfall zone* is located in Northern India, it is known as A1 zone (most arid zone or primary zone) and cover the territories of Western part of Rajasthan, and parts the states of Haryana and Gujarat. This zone has an annual rainfall level between 320 and 400 mm; its soil composition is sand and entisol (59%). On the other hand the *Higher rainfall zones* (A and B, being less arid than zone A1) are located in either the northern-central part of India or peninsular India. The A zone (secondary zone) comprises the northern and north western part of India including the eastern Rajasthan and parts of Haryana, Gujarat, and Uttar Pradesh; It has an annual rainfall level near to 400 mm with fine sand and entisol (31%) soil composition accounting low levels of organic matter content. The B zone (tertiary zone) comprises the Peninsular Indian states of Maharashtra, Tamil Nadu, and Karnataka; its annual rainfall level is among 400–520 mm and has heavy soil composition as entisol (28%) and alfisol (26%) (Manga and Kumar, 2011; Rai et al., 2015). In effect those differences between soil profile and rainfall intensity, and distribution in both zones may cause an effect on the crop adaptation and its breeding history.

Restricting transpiration under conditions of high evaporative demand is an important avenue to gain in efficiency of water use. During the last decade, so large genotypic variation in the restriction of water loss under high VPD has been found in different crop species (Reviewed in Vadez et al., 2014). How much the VPD-response depends on the environment where genotype/cultivars have evolved or for which they have been bred, is unknown. This trait is important because it leads to improved transpiration efficiency (TE). A restricted transpiration (lower TR) under high VPD in drought environments resulted in the increment of yield (Gholipoor et al., 2010; Aparna et al., under review). This trait is also hypothesized to be explained by differences in the hydraulic characteristics of the plant.

Another option for controlling water use is for plants exposed to progressive water stress to reduce transpiration at high soil moisture levels, expressed as the fraction of transpirable soil water (FTSW) remaining in the soil. The genotypes that are more sensitive to soil drying initiate the stomatal closure at higher soil water content, which contributes to conserving soil water (Sinclair and Rufty, 2012; Vadez et al., 2014). This genetic variability in this response has been observed in cereals like pearl millet (Kholová et al., 2010b), sorghum (Gholipoor et al., 2012; Choudhary et al., 2013) and also in legumes like chickpea (Zaman-Allah et al., 2011) or groundnut (Devi et al., 2010). Henceforth, those two aspects mentioned above, the sensitivity of the stomata under high VPD and soil drying, both contribute to a better conservation of soil water and may contribute to enhanced yields in scenarios with limited water (Sinclair, 2012). They are also supposed to enhance TE (Vadez et al., 2014).

Therefore, the aim of this investigation was to assess different traits controlling plant water use in hybrids that were bred specifically for agro-ecological zones with different rainfalls. Specifically, the transpiration response to increasing VPD and possible mechanisms explaining it, transpiration response to soil drying, and the leaf canopy development, were assessed. A comparison was also made of these traits between the hybrids and their parental lines.

## Materials and Methods

### Genetic Material and Location

The genotypes collection of pearl millet (*P. glaucum* L.) had been bred in two agro-ecological scenarios: lower and higher rainfall zones of India. We assessed in total 22 genotypes developed in Zone A1 (lower rainfall), 19 genotypes in Zone A, and 18 in Zone B (higher rainfall) among three experiments (see **Table 1**). In addition, 8 of these hybrids (4 from A1, 2 from A, and 2 from B zones) along with parental R- and B-lines were compared.

**Table 1:** Lists of Pearl Millet parental (B line and R line) and F_1_ hybrids tested in the transpiration response to VPD (Vapor Pressure Deficit) and Soil drying experiments.

Response to high VPD (Exp.1) and progressive soil drying (F_1_ hybrids, B line and R line)			Response to high VPD (Exp.2) (F_1_ hybrids)		Response to high VPD and growth outdoors (Exp.3 and Exp.4) (F_1_ hybrids)			
Genotype	Class	Zone	Genotype	Zone	Genotype	Zone	Genotype	Zone
HOPE 2013-AHT-R-8	F_1_	A1	HOPE-2014 AHT-R-15	A1	HOPE-2014 AHT-R-1	A1	AHT A/K14-2	A
96666 B	B	A1	HOPE-2014 AHT-R-7	A1	HOPE-2014 AHT-R-9	A1	AHT A/K14-3	A
RIB 3135/18	R	A1	HOPE-2014 AHT-R-11	A1	HOPE-2014 AHT-R-14	A1	IHT A2 /K14-24	A
HOPE-2013 AHT-R-14	F_1_	A1	HOPE 2013-AHT-R-8	A1	HOPE-2014 AHT-R-8	A1	AHT B/K14-22	A
843-22 B	B	A1	HOPE-2013 AHT-R-14	A1	HOPE-2014 AHT-R-16	A1	EMTT /K14-10	A
MRC S1-97-3-4-B-B-1-B-1-B	R	A1	HHB 67 imp	A1	HOPE-2014 AHT-R-17	A1	IHT A1 /K14-4	A
HOPE-2013 AHT-R-18	F_1_	A1	AHT II/K14-7	A	HOPE-2014 AHT-R-4	A1	IHT A2 /K14-13	A
(EERC-HS-29)-B-13-4-5-2	B	A1	AHT A/K14-5	A	HOPE-2014 AHT-R-15	A1	IHT B1 /K14-5	B
88004 B	R	A1	AHT II/K14-9	A	HOPE-2014 AHT-R-7	A1	IHT B1 /K14-20	B
HHB 67 imp	F_1_	A1	IHT A2 /K14-24	A	HOPE-2014 AHT-R-11	A1	AHT II/K14-14	B
843-22 B	B	A1	AHT A/K13-4	A	HOPE 2013-AHT-R-8	A1	AHT B/K14-20	B
H77/833-2-202	R	A1	AHT A/K13-5	A	HOPE-2013 AHT-R-14	A1	AHT II/K14-11	B
AHT A/K13-4	F_1_	A	IHT B1 /K14-20	B	HOPE-2013 AHT-R-18	A1	AHT II/K14-20	B
ICMB 97222	B	A	AHT II/K14-20	B	HHB 67 imp	A1	IHT B1 /K14-10	B
MRC HS-130-2-2-1-B-B-3-B-B-B-1-3-1	R	A	IHT B1 /K14-10	B	AHT II/K14-7	A	IHT B1 /K14-26	B
AHT A/K13-5	F_1_	A	AHT-II/K13-5	B	AEHT /K14-2	A	AHT II/K13-18	B
ICMB 04222	B	A	ICMH 1201	B	AHT II/K14-8	A	AHT II/K14-5	B
JBV 3 S1 -237-1-3-3-1-B	R	A	AHT-II/K13-24	B	AEHT /K14-18	A	AHT II/K13-5	B
AHT-II/K13-5	F_1_	B			AHT A/K14-5	A	AHT II/K13-6	B
ICMB 99222	B	B			AHT II/K14-9	A	ICMH 1201	B
ICMV 96490-S1-15-1-2-1-1	R	B						
AHT-II/K13-24	F_1_	B						
ICMB 98222	B	B						
(MC 94 C2-S1-3-2-2-2-1-3-B-B xAIMP 92901 S1-488-2-1-1-4-B-B)-B-2-2-2	R	B						

*Lower (A1) and higher (A and B) rainfall zones.*

The first glasshouse experiment (Exp.1) was an assessment of the transpiration rate response (TR) to increasing evaporative demand (vapor pressure deficit, VPD) and to soil drying response. This experiment was conducted in 2014 with (24 genotypes), i.e., eight combinations of F_1_ hybrids with their parental B line (male sterile) and R line (restorer); four combinations were bred for the lower rainfall zone (A1), and other four combinations were bred for the higher rainfall zones with half of them for zone A and the other half for zone B (**Table 1**). In the same way during 2015, a second experiment in glasshouse (Exp.2) of transpiration rate response to evaporative demand was conducted with 18 F_1_ hybrids: 6 were bred for the lower rainfall zone (A1) and 12 were bred for the zones A and B (see **Table 1**). Furthermore, two additional experiment (Exp.3 and Exp.4) were conducted outdoors in 2015 and 2016, respectively, at the LeasyScan facility (Vadez et al., 2015) at ICRISAT with a larger number of F_1_ hybrids (40 genotypes): 14 of them were bred for the lower rainfall zone (A1) and 26 were bred for the higher rainfall zones: 13 belonged to zone A and other 13 to zone B (**Table 1**). In Exp.3 the transpiration response to VPD was assessed in the LeasyScan platform under natural VPD increases. The purpose of Exp.3 and Exp.4 were to compare the canopy development of these hybrids, along with an assessment of the transpiration rate to natural increase in VPD (Exp.3). In Exp.3, the daily average temperature and relative humidity (RH) range was 22–28°C and 34–84%, respectively, while in Exp.4 the temperature and RH range was 26–31°C and 30–69%, respectively. All the experiments were conducted during February–April season of 2014 and 2015, and the soil used was sandy claim loam Alfisol which availability water content was ∼10% and bulk density of 1.5 g/cc. The soil was fertilized with di-ammonium phosphate (DAP) at a rate of 0.3 g/kg. All experiments were located at the ICRISAT campus in Patancheru (India): latitude 17°30′N; longitude 78°16′E; altitude 549 m.

### Transpiration Response to Vapor Pressure Deficit (VPD) in Controlled Conditions

Exp.1 and Exp.2 were carried out in controlled conditions, with five biological replicates per genotype (*n*_1_ = 120 and *n*_2_ = 90). All plants were sown in 8 Kg pots filled with red soil and grown in glasshouse (17–35°C/65–35 %RH). Ten to 15 days after sowing, each pot was thinned to a single plant. The pots were watered every 1–3 days with soft water and plants were grown for 30 days before the experiment started (Vegetative stage: Zadocks scale 24–26, depending of each genotype). One day before the TR experiment, all pots were watered and allowed to drain overnight to reach soil capacity in the pot; the following morning each pot was covered with a plastic sheet and a layer of plastic beads to minimize soil evapotranspiration. After that, the pots were transferred to a Conviron E-15 (Controlled Environments, Winnipeg, MB, Canada) growth chamber for acclimatization. The next day, the TR response to high VPD was performed in the chamber by exposing the plants organized in a complete randomized design to a controlled ladder of increasing VPD, applied by changing both temperature and humidity every hour from 7 am (23°C/80 %RH) up to 4 pm (40°C/45 %RH), at a constant light flux of ∼450 μmoles m^-2^ s^-1^. Plant transpiration was measured by weighing pots every hour in a bench electronic 10 kg balance with a resolution of 0.1 g (FBK, Kern & Sohn GmbH, Balingen, Germany), giving one transpiration value per plant at each VPD point. To avoid the plant size variation, in each plant the transpiration was normalized by its leaf area (LA), this normalization is the rate between transpiration per unit of time divided by total LA. After the last recorded weight, the plants were harvested by cutting the stem above 2 cm of the soil level, and the xylem exudate was collected immediately in 11-mL pre-weighted tubes containing cotton inside during 20 min, after that the tubes were closed and their weight was recorded. Subsequent, the LA was measured with a LA meter (LA meter LI3000 model, Li-Cor, Lincoln, NE, United States), and finally the stem and leaves were dried at 60°C in an oven during 72 h. The following day, the roots were carefully washed and the measurement of root length (RL) was conducted using the scanning equipment and imaging software WinRizho (WinRizho^TM^ Pro, Regent Instruments Inc., Quebec City, QC, Canada).

### Transpiration Response to Evaporative Demand Outdoors

A transpiration rate response to naturally increasing VPD conditions was performed outdoors during February-March 2015 (Exp.3), this period of the year is known to enjoy high temperature and low RH%, giving a high VPD condition. Six biological replicates per genotypes (*n*_3_ = 240) and additional six pots without plant to estimate the evapotranspiration of bare soil; In brief, the platform is a laser scanner-based technique (PlantEye F300, Phenospex, Heerlen, Netherlands) providing 3D point clouds from which plant parameters, including LA, are measured every 2 h (Vadez et al., 2015). The temperature and RH (20–39°C/20–70 RH% range) were recorded each 30 min (Campbell Scientific, Logan, UT, United States). The seeds were sown in 15 kg pots filled with red soil; 12 days after, each pot was thinned leaving two plants per pot. One experimental unit consisted of two such pots, i.e., four plants per experimental unit. The pots were automatically watered every 1–3 days with soft water, the plants were grown for 34 days before the experiment started (vegetative stage: Zadocks scale 28–30, depending of each genotype). The day before the transpiration assessment, each pot was over-watered with 1 L of soft water by the afternoon and let for drainage overnight. The transpiration assay was carried out over two consecutive days, by weighing each pot in an electrical 20 kg balance with a resolution of 0.1 g (FBK, Kern & Sohn GmbH, Balingen, Germany) at three time points during the day: 6:30 am, 10:00 am and 3:00 pm. After the last weighing of the afternoon of the 1st day, all the plants were watered with 1 L of soft water again, drained overnight and the next day the same weighing procedure was repeated. At the end of the 2nd day all plants were harvested and dried during 72 h at 60°C in an oven similarly to the experiments described above. The environmental temperature range was 21.8–39.4°C and the relative humidity range was 21–67 RH%, leading to a range of VPD values of 0.8–5.9 kPa during the time frame of the experiment. Based on this, the transpiration recorded between the first two time points [6:30 am (0.8 kPa)–10:00 am (3.4 kPa)] was considered to correspond to a low to mild VPD period, whereas the transpiration in the second period [until 3:00 pm (5.3 kPa)] was considered to take place during high VPD conditions. The LA 3D data was extracted from the platform database to calculate the transpiration rate (unit) for each day of experiment. The relationship between the measured and scanned LA was validated with the reported transformation LA3d = 0.22LA + 241 (Vadez et al., 2015), where 3D LA is the area measured by the scanner and LA was the observed LA measured with Li 3000 LA meter. Later the transpiration rate was calculated after estimating the soil evaporation from the non-sown pots. To do so, it was considered that soil evaporation was maximum at a leaf area index (LAI) of zero, and nil at a LAI of 2. In between these boundary LAI values, soil evaporation was considered to be proportional to the LAI. Transpiration rate was then calculated by dividing transpiration values by the LA. To fit the data of TR and VPD levels, we applied a linear regression, and then the slopes were compared among the genotypes. The growth outdoors (Exp.3 and Exp.4) was evaluated with LA3d and plant height data generated by the phenospex platform. Temperature data were used to convert days after sowing data into equivalent days at 20°C to compare growth curves between both seasons, following earlier work (Parent and Tardieu, 2012).

### Transpiration Response to Soil Drying

The dry-down experiment was conducted in the glasshouse with semi-regulated temperature and humidity (17–35°C/65-35 %RH) during February–March 2014 with the same plant material used in Exp.1 with 10 biological replicates for each genotype (*n*_4_ = 240). The seeds were sown in 8 kg pots filled with Alfisol, after 10–12 days all pots were thinned to one single plant per pot. The plants grew under fully irrigated conditions during 30 days (Zadock stage: 26–32, depending on the genotype).

The afternoon before the dry-down started all pots were irrigated with soft water to soil capacity, let to drain overnight and covered with a plastic sheet and a layer of plastic beads to avoid water loss by evaporation. The next morning all pots were weighed and this measure was recorded as the initial weight at field capacity. Then five replicates of each genotype were assigned to a well-watered treatment (WW), in which transpiration was replenished every day; the other five replicates were assigned to water-deficit treatment (WS) with an irrigation regime that allowed a maximal transpiration water loss on each day, by replenishing water in excess of this allowed maximum. This procedure allowed similar kinetics of stress imposition to plants varying in size. All pots were weighed every morning (10:00 am), their daily transpiration was calculated, and each pot was irrigated according to its water regime. This procedure was maintained until the transpiration of the WS plants fell below 10% of that in their WW controls. Then, plants were harvested, the LA was measured in the WW plants, and dry weight measured after drying samples in an oven at 60°C during 72 h.

The Fraction of Transpirable Soil Water (FTSW), as a soil water stress indicator, and the Normalized Transpiration Ratio (NTR) were calculated. First, the transpiration ratio (TR) of all plants was calculated by dividing each transpiration value by the mean of the transpiration of the WW plants, within each genotype. Then, to avoid variations on individual plant size a second normalization consisted of dividing TR values by an average of the TR obtained during the first 5 days, i.e., before any water stress occurred. Therefore, NTR values were centered on 1.0 during the well-watered period before the stress started in the soil, and then started decreasing from 1.0 when stress started. The drydown was over for a given genotype when the NTR value fell below 0.1, i.e., when transpiration of the WS plants fell below 10% of that in their WW controls. The change of NTR was plotted against the FTSW, the FTSW was expressed as the volumetric water content of the soil, it was calculated using the following equation: (daily weight–final weight)/(initial weight–daily weight). To fit the data plotted as NTR against FTSW, we applied a two-segment linear regression, and then the slope and the FTSW threshold were compared among the genotypes.

### Data Analysis

The multivariate analysis of the data was performed with all data of the experiments performed in this study. The principal component analysis (PCA) was performed in R by reducing the dimensions of the trait variables to differ both transpiration and physiological response between higher and lower rainfall, separate PCA were performed for each rainfall zone, two analyses for traits of Exp.1 to TR response to high VPD and other two analyses for the TR response to soil drying.

The statistical analysis of data for the TR response to increasing VPD in Exp.1 and Exp.2, and of data plotted as NTR against FTSW in the dry down experiment, was done by Segmental non-linear regression and Linear regression [(*Y*_1_ = slope1.X + intercept 1 and *Y*_2_ = slope2.X + intercept2) or Linear regression (*Y*_1_ = slope1.X + intercept 1)]. Both regressions with best fitting curve model with 1000 iterations and parameter comparisons, and One-way ANOVA followed by Dunnett’s multiple comparisons. The growth comparison in Exp.3 and Exp.4 were performed with Sigmoidal and linear regression fit comparisons (*p* < 0.05) for the LA and plant height curves, all tests were performed with the provider considerations using GraphPad Prism (version 7.00 for Windows, GraphPad Software, La Jolla, CA, United States^1^). The analysis of the TR response outdoors plotting normalized TR against VPD in Exp.3, and the physiological parameters of all experiments were done by Analysis of Variance (ANOVA) test, Student *t*-test, LSD (least significant differences) test, Linear regression, Pearson Correlation, and PCA, all tests were performed using the provider indications with the Linear model tool in Stats R package (Core Team 2015). In all analyses the data was considered as significant are *p* < 0.05 and all data shown in the tables are means and SEM.

## Results

### F1 Hybrid Response to High VPD and to Progressive Soil Drying

**FIGURE 1:**
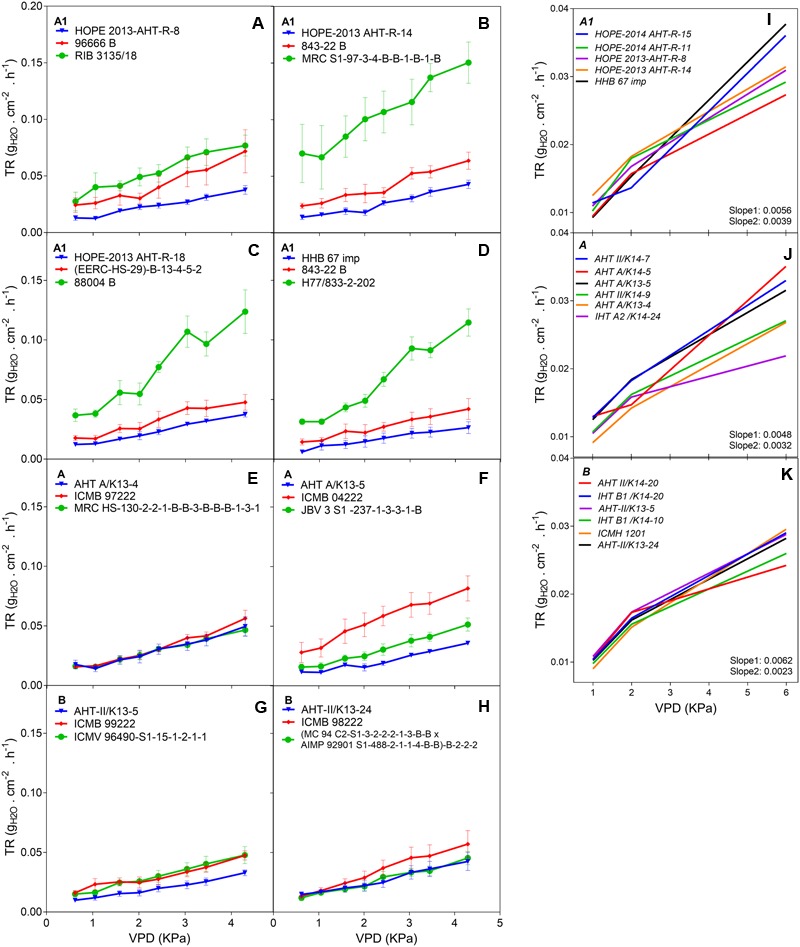
Transpiration response to high VPD of the combinations F1-Hybrids and parental (Exp.1 and Exp.2) genotypes bred for lower (A1) and higher (A and B) rainfall zones of India. **(A–D)** Show the response of the combinations [F_1_ hybrids (blue), B line or male-sterile (red) and the R line or restorer (green)] bred in lower rainfall zone (A1); **(E–H)** show the response of higher rainfall genotypes bred in A and B zones, respectively. Each curve shows a set of points with standard error. **(I–K)** Show the linear regression of the response to high VPD from hybrids bred in zones A1, A and B, respectively. VPD, vapor pressure deficit; TR, Transpiration rate.

The F_1_ hybrid response to high VPD in glasshouse showed seasonal slopes variation. In Exp.1 all groups (A1, A and B) had similar average slopes under low VPD (*slope1:* 0.0053*_Exp.1_* and 0.0055*_Exp.2_*) but different average slopes under high VPD (*slope2:* 0.0078*_Exp.1_* and 0.0033*_Exp.2_*). There was a variation in the slope of the TR response to increasing VPD levels, under low VPD across Exp.1 (**Figures 1A–H**) and Exp.2 (**Figures 1D,I–K**). Across years, the TR response under low VPD (slope 1) of low and high rainfall hybrids was similar (0.0057, A1 zone – 0.0048, A zone – 0.0059, B zone). By contrast, across both years in those glasshouse experiments, the TR response under high VPD (*slope 2*) was higher in lower rainfall hybrids (0.0054) than in the hybrids from the B zone (0.0036 – *p* < 0.05) (**Figure 1** and **Table 2**). Nevertheless, when the transpiration response to increasing VPD was measured in plants grown under high VPD outdoors condition, F_1_ hybrid bred for higher rainfall and hybrids bred for lower rainfall had similar slopes (**Table 3**), suggesting an effect of the plant growth environment on the VPD response.

**Table 2:** Slopes of the response to high VPD of pearl millet F_1_ Hybrids from lower rainfall zone (A1) and higher rainfall zones (A and B) of India.

	Hybrids (F_1_)	Zone	Slope 1			Slope 2		
Lower rainfall	HOPE-2014 AHT-R-15	A1	0.0022	±	0.0002	0.0056	±	0.0000
	HOPE-2014 AHT-R-7	A1	0.0063	±	0.0001	0.0029	±	0.0000
	HOPE-2014 AHT-R-11	A1	0.0077	±	0.0000	0.0028	±	0.0000
	HOPE 2013-AHT-R-8	A1	0.0058	±	0.0001	0.0035	±	0.0000
	HOPE-2013 AHT-R-14	A1	0.0057	±	0.0000	0.0033	±	0.0000
	HHB 67 imp	A1	0.0060	±	0.0000	0.0056	±	0.0000
	^∗^HOPE 2013-AHT-R-8	A1	0.0070	±	0.0019	0.0068	±	0.0012
	^∗^HHB 67 imp	A1	0.0061	±	0.0031	0.0050	±	0.0019
	^∗^HOPE-2013 AHT-R-14	A1	0.0044	±	0.0022	0.0103	±	0.0014
	^∗^HOPE-2013 AHT-R-18	A1	0.0060	±	0.0016	0.0082	±	0.0010

Higher rainfall	AHT II/K14-7	A	0.0054	±	0.0001	0.0037	±	0.0000
	AHT A/K14-5	A	0.0017	±	0.0002	0.0051	±	0.0000
	AHT II/K14-9	A	0.0055	±	0.0003	0.0027	±	0.0001
	IHT A2 /K14-24	A	0.0053	±	0.0001	0.0015	±	0.0000
	AHT A/K13-4	A	0.0051	±	0.0000	0.0032	±	0.0000
	AHT A/K13-5	A	0.0059	±	0.0002	0.0033	±	0.0000
	^∗^AHT A/K13-4	A	0.0064	±	0.0040	0.0108	±	0.0025
	^∗^AHT A/K13-5	A	0.0036	±	0.0013	0.0086	±	0.0008
	
	IHT B1 /K14-20	B	0.0061	±	0.0000	0.0031	±	0.0000
	AHT II/K14-20	B	0.0069	±	0.0002	0.0017	±	0.0000
	IHT B1 /K14-10	B	0.0058	±	0.0001	0.0026	±	0.0000
	AHT-II/K13-5	B	0.0065	±	0.0001	0.0029	±	0.0000
	ICMH 1201	B	0.0061	±	0.0001	0.0036	±	0.0000
	AHT-II/K13-24	B	0.0059	±	0.0001	0.0030	±	0.0000
	^∗^AHT-II/K13-5	B	0.0046	±	0.0021	0.0069	±	0.0013
	^∗^AHT-II/K13-24	B	0.0054	±	0.0025	0.0056	±	0.0017
	A1 zone_average_		**0.0057^a^**	**±**	**0.009**	**0.0054^a^**	**±**	**0.0005**
	A zone_average_		**0.0048^a^**	**±**	**0.0007**	**0.0048^ab^**	**±**	**0.0004**
	B zone_average_		**0.0059^a^**	**±**	**0.0006**	**0.0036^b^**	**±**	**0.0003**

*The table shows the TR slope variation of the genotypes under low VPD (Slope 1) and high VPD (Slope 2) assayed in greenhouse during the Exp.1 and Exp.2 fitting a Segmented linear model (all are significant at *P* < 0.001) and *T*-test (*p < 0.05*). Means of five replicates and SE are shown, superscript letters indicate the mean separation.*

**Table 3:** Transpiration response of F_1_ hybrid assayed outdoors (Exp.3).

	Genotype	Zone	Slope			Intercept		
Lower rainfall	HOPE-2014 AHT-R-17	A1	0.0164	±	0.0014	**-**0.0427	±	0.0064
	HOPE-2014 AHT-R-11	A1	0.0161	±	0.0013	**-**0.0426	±	0.0057
	HOPE-2014 AHT-R-4	A1	0.0161	±	0.0015	**-**0.0409	±	0.0066
	HOPE-2013 AHT-R-14	A1	0.0157	±	0.0018	**-**0.0401	±	0.0079
	HOPE 2013-AHT-R-8	A1	0.0154	±	0.0014	**-**0.0434	±	0.0064
	HOPE-2014 AHT-R-1	A1	0.0146	±	0.0013	**-**0.0383	±	0.0056
	HOPE-2014 AHT-R-16	A1	0.0145	±	0.0022	**-**0.0385	±	0.0101
	HOPE-2014 AHT-R-9	A1	0.0140	±	0.0015	**-**0.0370	±	0.0065
	HOPE-2014 AHT-R-7	A1	0.0132	±	0.0014	**-**0.0328	±	0.0061
	HOPE-2014 AHT-R-8	A1	0.0130	±	0.0004	**-**0.0324	±	0.0019
	HOPE-2014 AHT-R-14	A1	0.0125	±	0.0015	**-**0.0323	±	0.0064
	HOPE-2013 AHT-R-18	A1	0.0117	±	0.0009	**-**0.0278	±	0.0040
	HOPE-2014 AHT-R-15	A1	0.0112	±	0.0013	**-**0.0231	±	0.0058
		*A1_average_*	**0.0140^a^**	**±**	**0.0004**	**-0.0356^a^**	**±**	**0.0020**

Higher rainfall	AEHT/K14-2	A	0.0170	±	0.0023	-0.0450	±	0.0099
	AHT A/K14-2	A	0.0154	±	0.0015	**-**0.0444	±	0.0065
	AHT II/K14-9	A	0.0150	±	0.0014	**-**0.0411	±	0.0061
	AHT II/K14-8	A	0.0139	±	0.0011	**-**0.0361	±	0.0050
	AHT A/K14-3	A	0.0137	±	0.0013	**-**0.0322	±	0.0056
	AHT B/K14-22	A	0.0135	±	0.0014	**-**0.0331	±	0.0061
	EMTT/K14-10	A	0.0133	±	0.0013	**-**0.0346	±	0.0057
	IHT A2/K14-24	A	0.0130	±	0.0006	**-**0.0332	±	0.0027
	AHT A/K14-5	A	0.0123	±	0.0025	**-**0.0311	±	0.0112
	AEHT/K14-18	A	0.0121	±	0.0012	**-**0.0312	±	0.0053
	IHT A1/K14-4	A	0.0117	±	0.0013	**-**0.0272	±	0.0057
	AHT II/K14-7	A	0.0116	±	0.0013	**-**0.0269	±	0.0062
		*A*_average_	**0.0128^a^**	**±**	**0.0004**	**-0.0317^ab^**	**±**	**0.0018**
	IHT B1/K14-5	B	0.0171	±	0.0012	**-**0.0460	±	0.0052
	AHT II/K14-14	B	0.0171	±	0.0012	**-**0.0460	±	0.0052
	AHT II/K14-20	B	0.0158	±	0.0011	**-**0.0430	±	0.0049
	AHT II/K13-5	B	0.0152	±	0.0017	**-**0.0396	±	0.0073
	AHT II/K13-18	B	0.0146	±	0.0006	**-**0.0389	±	0.0028
	ICMH 1201	B	0.0141	±	0.0014	**-**0.0348	±	0.0062
	AHT II/K14-5	B	0.0141	±	0.0020	**-**0.0403	±	0.0092
	AHT B/K14-20	B	0.0138	±	0.0007	**-**0.0340	±	0.0030
	IHT B1/K14-20	B	0.0135	±	0.0014	**-**0.0336	±	0.0061
	AHT II/K13-6	B	0.0128	±	0.0011	**-**0.0285	±	0.0051
	IHT B1/K14-26	B	0.0126	±	0.0025	**-**0.0288	±	0.0106
	AHT II/K14-11	B	0.0115	±	0.0017	**-**0.0211	±	0.0077
	IHT B1/K14-10	B	0.0106	±	0.0015	**-**0.0188	±	0.0070
		*B*_average_	**0.0130^a^**	**±**	**0.0004**	**-0.0309^b^**	**±**	**0.0019**
	Lower rainfall_average_		**0.0140^a^**	**±**	**0.0004**	**-0.0356^a^**	**±**	**0.0020**
	Higher rainfall_average_		**0.0129^a^**	**±**	**0.0004**	**-0.0313^a^**	**±**	**0.0018**

*The table shows the slopes variation under high VPD of hybrids bred for lower rainfall zone (A1) and higher rainfall zones (A and B) fitted in a linear regression (all are significant at *P* < 0.001) analysed with LSD test (*p* < 0.05). Means of six replicates and SE are shown, superscript letters indicate the mean separation.*

**FIGURE 2:**
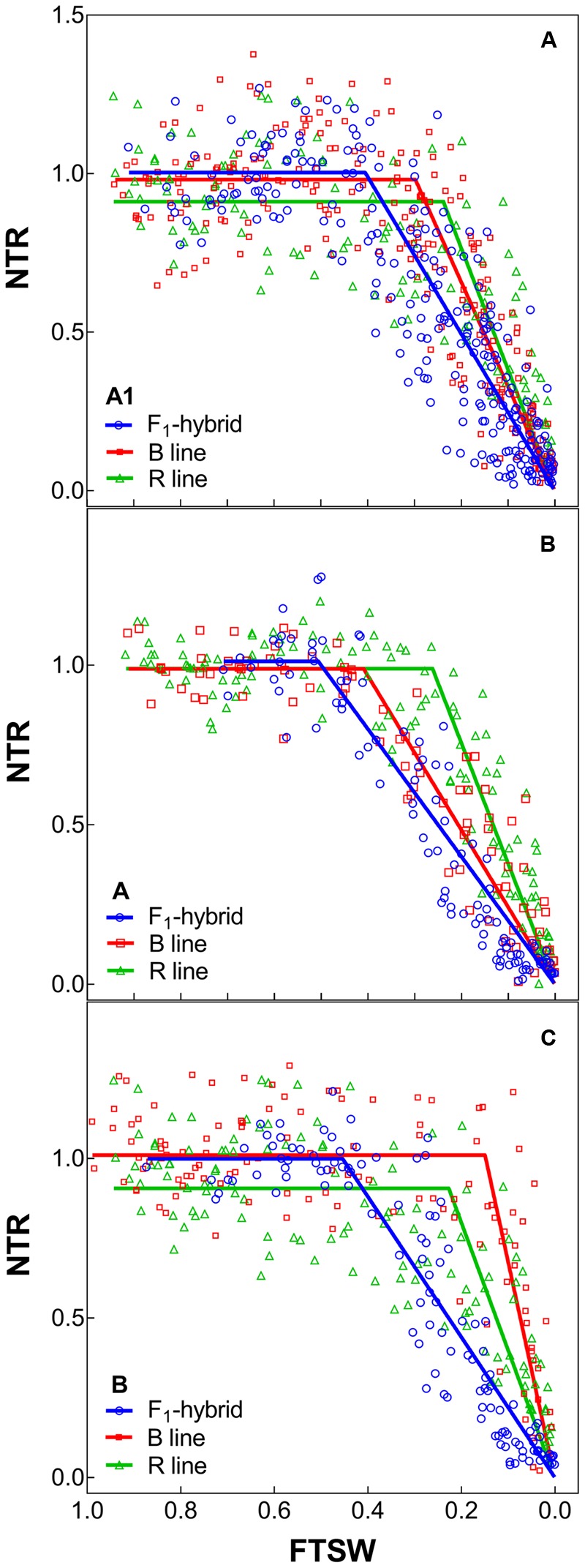
Transpiration response to soil drying of the combinations F_1_ Hybrids and parental that were bred for lower (A1) and higher rainfall zones (B and C) of India. **(A–C)** Shows dry down response of combinations [F_1_ hybrids (blue), B line or male-sterile (red) and the R line or restorer (green)] bred in zone A1, A and B, respectively. Each biological replicate (circle) and its segmented regression line are represented. NTR, normalized transpiration rate; FTSW, fraction of transpirable soil water.

With regards to the transpiration response to progressive soil drying, the F_1_ hybrids from high rainfall and low rainfall zones grown in glasshouse had a similar behavior (**Figure 2**: blue lines). All showed a water conservative behavior with FTSW threshold that were relatively high, i.e., around 0.44–0.47 (see **Table 4**), and declining slopes not showing any significant difference.

**Table 4:** Transpiration response to the progressive soil drying of F_1_ Hybrid evolved in lower rainfall zone (A1) and higher rainfall zones (A and B).

	Hybrid	Zone	Slope	FTSW Threshold	Response
Higher rainfall	HOPE-2013 AHT-R-18	A1	2.67 ± 0.24	0.32 ± 0.02	Less conservative
	HOPE 2013-AHT-R-8	A1	2.29 ± 0.13	**0.44** ± 0.02	Conservative
	HHB 67 imp	A1	2.15 ± 0.14	**0.50** ± 0.03	Conservative
	HOPE-2013 AHT-R-14	A1	1.72 ± 0.13	**0.52** ± 0.03	Conservative
		A1_average_	2.21^a^ ± 0.16	0.44^a^ ± 0.03	

Lower rainfall	AHT A/K13-5	A	2.20 ± 0.14	**0.45** ± 0.03	Conservative
	AHT A/K13-4	A	2.15 ± 0.09	**0.51** ± 0.02	Conservative
		*A*_average_	2.17^a^ ± 0.11	0.48^a^ ± 0.02	
	AHT-II/K13-5	B	2.20 ± 0.11	**0.46** ± 0.02	Conservative
	AHT-II/K13-24	B	2.21 ± 0.12	**0.46** ± 0.02	Conservative
		*B*_average_	2.20^a^ ± 0.11	0.46^a^ ± 0.02	
	Lower rainfall_average_		**2.21^a^ ± 0.16**	**0.44^a^ ± 0.03**	**Conservative**
	Higher rainfall_average_		**2.18^a^ ± 0.11**	**0.47^a^ ± 0.02**	**Conservative**

*Differences analysed by LSD test (*p* < 0.05), the superscript letters indicate the separation of means. FTSW represents the fraction of transpirable soil water and the FTSW threshold is the FTSW at which the transpiration of plants exposed to water stress began to decline in comparison to fully irrigated controls. The last column provides a qualitative assessment of the response to soil drying, and the conservative FTSW values are indicated in bold.*

### Responses to High VPD and Progressive Soil Drying of the Combinations of F1 Hybrid and Parental Lines B and R

The profile of the combinations (F_1_ hybrids and parental) shown in **Table 5** indicated that F_1_ hybrids adapted in both rainfall zones had lower declining NTR slopes and lower FTSW threshold than their parents (**Figure 2** and Supplementary Figure S1). This relation was confirmed with the PCA analysis (see below), and this may have reflected their heterotic vigor. The difference in the slope of the transpiration response to increasing VPD in the R lines of the A1 zone was about two fold compared to the B lines and three fold compared to the hybrids.

**Table 5:** Transpiration response to high VPD and soil drying (Exp.1) among combinations of F_1_ hybrids and parental lines (R and B) bred for lower (A1) and higher rainfall zones (A and B) of India.

	Genotype	Zone	Class	Response to high VPD						Response to soil drying					
				Slope 1			Slope 2			NTR slope			FTSW threshold		
Lower	HOPE 2013-AHT-R-8	A1	F_1_	**0.0070**	**±**	**0.0019**	**0.0068**	**±**	**0.0012**	**2.29**	**±**	**0.13**	**0.44**	**±**	**0.02**
rainfall	96666 B	A1	B	0.0062	±	0.0060	0.0092	±	0.0039	2.31	±	0.30	0.38	±	0.04
	RIB 3135/18	A1	R	0.0143	±	0.0071	0.0131	±	0.0044	4.77	±	0.54	0.20	±	0.02
	HOPE-2013 AHT-R-14	A1	F_1_	**0.0044**	**±**	**0.0022**	**0.0103**	**±**	**0.0014**	**1.72**	**±**	**0.13**	**0.52**	**±**	**0.03**
	843-22 B	A1	B	0.0078	±	0.0045	0.0133	±	0.0028	3.03	±	0.19	0.30	±	0.01
	MRC S1-97-3-4-B-B-1-B-1-B	A1	R	0.0207	±	0.0189	0.0312	±	0.0118	–		–	–		–
	HOPE-2013 AHT-R-18	A1	F_1_	**0.0060**	**±**	**0.0016**	**0.0082**	**±**	**0.0010**	**2.67**	**±**	**0.24**	**0.32**	**±**	**0.02**
	(EERC-HS-29)-B-13-4-5-2	A1	B	0.0090	±	0.0042	0.0094	±	0.0026	2.68	±	0.15	0.35	±	0.02
	88004 B	A1	R	0.0199	±	0.0085	0.0282	±	0.0053	3.40	±	0.29	0.25	±	0.02
	HHB 67 imp	A1	F_1_	**0.0061**	**±**	**0.0031**	**0.0050**	**±**	**0.0019**	**2.15**	**±**	**0.14**	**0.50**	**±**	**0.03**
	843-22 B	A1	B	0.0081	±	0.0036	0.0042	±	0.0024	2.71	±	0.28	0.35	±	0.03
	H77/833-2-202	A1	R	0.0180	±	0.0055	0.0285	±	0.0034	4.51	±	0.44	0.19	±	0.01

Higher	AHT A/K13-4	A	F_1_	**0.0064**	**±**	**0.0040**	**0.0108**	**±**	**0.0025**	**2.15**	**±**	**0.09**	**0.51**	**±**	**0.02**
rainfall	ICMB 97222	A	B	0.0071	±	0.0027	0.0134	±	0.0017	2.07	±	0.11	0.46	±	0.02
	MRC HS-130-2-2-1-B-B-3-B-B-B-1-3-1	A	R	0.0077	±	0.0022	0.0093	±	0.0014	2.90	±	0.24	0.30	±	0.02
	AHT A/K13-5	A	F_1_	**0.0036**	**±**	**0.0013**	**0.0086**	**±**	**0.0008**	**2.20**	**±**	**0.14**	**0.45**	**±**	**0.03**
	ICMB 04222	A	B	0.0189	±	0.0073	0.0128	±	0.0046	1.99	±	0.20	0.42	±	0.03
	JBV 3 S1 -237-1-3-3-1-B	A	R	0.0076	±	0.0032	0.0114	±	0.0020	3.60	±	0.25	0.26	±	0.01
	AHT-II/K13-5	B	F_1_	**0.0046**	**±**	**0.0021**	**0.0069**	**±**	**0.0013**	**2.20**	**±**	**0.11**	**0.46**	**±**	**0.02**
	ICMB 99222	B	B	0.0047	±	0.0032	0.0092	±	0.0020	3.78	±	0.46	0.22	±	0.02
	ICMV 96490-S1-15-1-2-1-1	B	R	0.0094	±	0.0027	0.0062	±	0.0018	1.58	±	0.13	0.50	±	0.03
	AHT-II/K13-24	B	F_1_	**0.0054**	**±**	**0.0025**	**0.0056**	**±**	**0.0017**	**2.21**	**±**	**0.12**	**0.46**	**±**	**0.02**
	ICMB 98222	B	B	0.0126	±	0.0055	0.0120	±	0.0035	6.00	±	0.58	0.16	±	0.01
	(MC 94 C2-S1-3-2-2-2-1-3-B-Bx AIMP 92901 S1-488-2-1-1-4-B-B)-B-2-2-2	B	R	0.0077	±	0.0029	0.0095	±	0.0018	5.10	±	0.68	0.16	±	0.02

Lower rainfall_average_		F_1_		**0.0059^b^**	**±**	**0.0022**	**0.0076^b^**	**±**	**0.0014**	**2.21^c^**	**±**	**0.16**	**0.44^a^**	**±**	**0.02**
		B line		0.0078^ab^	±	0.0046	0.0090^a^	±	0.0029	2.68^b^	±	0.23	0.34^b^	±	0.02
		R line		0.0182^a^	±	0.0100	0.0252^a^	±	0.0062	4.23^a^	±	0.42	0.21^b^	±	0.02
Higher rainfall_average_		F_1_		**0.0050^a^**	**±**	**0.0025**	**0.0080^a^**	**±**	**0.0016**	**2.19^b^**	**±**	**0.12**	**0.47^a^**	**±**	**0.02**
		B line		0.0108^a^	±	0.0047	0.0118^a^	±	0.0029	3.46^a^	±	0.34	0.31^b^	±	0.02
		R line		0.0081^a^	±	0.0027	0.0091^a^	±	0.0017	3.30^a^	±	0.32	0.30^b^	±	0.02

*The table shows the slopes variation under high (Slope 2) and low (Slope 1) VPD, the FTSW threshold where the normalized transpiration rate (NTR) started to decline and the slope of the NTR decline upon soil drying beyond the FTSW threshold, the F1 hybrids are indicated in bold. Differences were analyzed by LSD test (*p* < 0.05), values shown as mean and SE, and the superscript letters indicate the mean separation. FTSW, fraction of transpirable soil water; NTR, normalized transpiration.*

### Physiological Parameters in F1 Hybrids of Higher and Lower Rainfall Zones

In the glasshouse experiments, having low VPD growth conditions (**Table 5**), F_1_ hybrids from higher rainfall showed significantly higher LA, exudation rate, root/shoot ratio, leaf thickness (SLA), and dry matter (TDM) than the ones from lower rainfall zones, but smaller RL (Supplementary Table S3). The exudation rate, or the exudation rate normalized by RL and root dry weight (RDW) were significantly larger for high rainfall zone hybrids than for the low rainfall zone hybrids. It should be noticed that for some of these parameters, there were also differences between the hybrids of the two higher rainfall zones.

**FIGURE 3:**
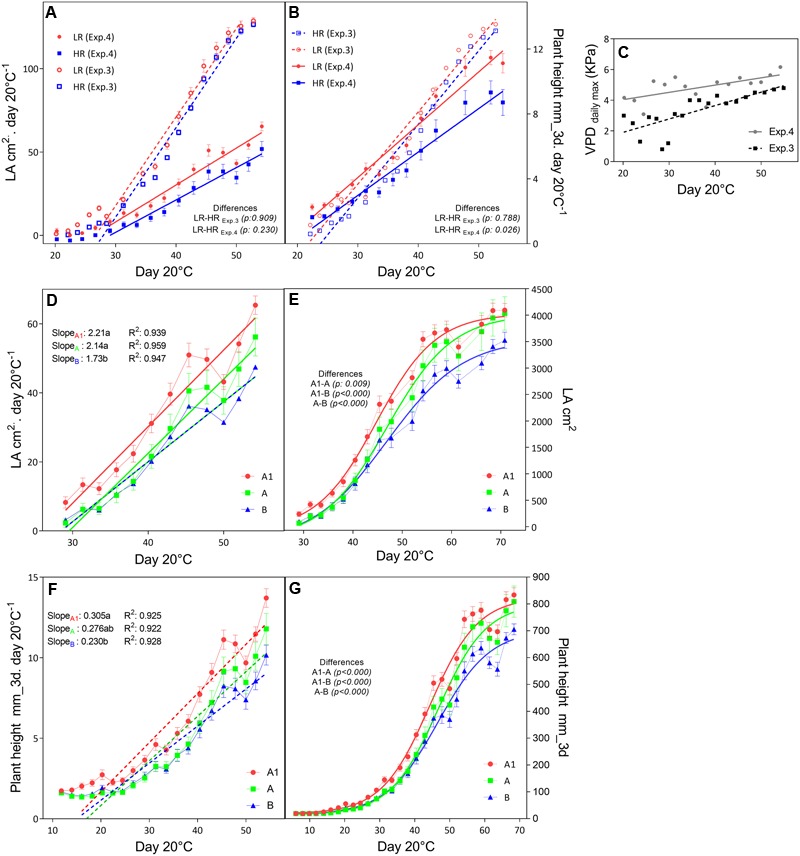
Growth development of F_1_ hybrids bred for higher (HR) and lower rainfall (LR) zones. The upper panels show the comparison of increases in leaf area **(A)** and plant height **(B)** per unit of days at 20°C within higher (HR, blue) and lower (LR, red) rainfall zones within two consecutive years (Exp.3 and Exp.4) where the maximum VPD levels **(C)** were different. The mid panel the development of leaves as the daily increase in LA **(D)** and the total LA per day at 20°C **(E)**. Similarly the bottom panels show the daily increase **(F)** and total plant height **(G)** measured by the scanner (mm). Comparisons of slopes (*p* < 0.05) were performed by linear regression of daily increase rates and are indicated with letters; and Sigmoidal regression were performed to compare total growth curves (*p* < 0.05). Differences are indicated.

In the outdoors experiments (Exp.3 and Exp.4), having high VPD conditions, only in Exp.3 the total dry matter and tiller numbers were higher in the low rainfall than in the high rainfall hybrids (**Table 6**), and the plants showed higher LA development in Exp.3 than in Exp.4 (**Figure 3A**), while in both experiment they reached a similar plant height at the exponential growth phase (**Figure 3B**). Throughout the crop development phase that was measured in the different experiments, the VPD conditions were higher in Exp.4 than in Exp.3 (**Figure 3C**). The daily increase in 3D LA was fitted to a linear regression as a function of days at 20°C and the slope of that regression was higher in the A1 zone hybrids than in the B-zone hybrids (**Figure 3D**). Similarly, the daily increase in plant height was fitted to a linear regression as a function of days at 20°C and the slope of that regression was higher in the A1 zone hybrids than in the B-zone hybrids (**Figure 3F**). As a consequence, hybrids bred in low rainfall zones had larger area and were taller than the high rainfall hybrids (**Figures 3E,G**) in this outdoor experiment under high VPD.

**Table 6:** Physiological parameters comparisons between F1 hybrids bred for the lower (A1) and higher (A and B) rainfall zones, grown in glasshouse (upper section) and outdoors (bottom section).

	F_1_ Hybrid	LA	TDM	SLA	Root shoot	Ex	RDW	RL	Ex-RL	Ex-RDW	DM control	DM stress
	Zone	(cm^2^)	(g)	(cm^2^.g^-1^)		(g.h^-1^)	(g)	(cm)	(g.h^-1^.cm^-1^)	(g.h^-1^.g^-1^)	(g)	(g)
Glasshouse	A1	700.39^c^	2.52^a^	218.82^b^	0.29^b^	0.15^b^	0.71^b^	114.84^a^	0.0018^a^	0.28^b^	35.20^b^	25.67^b^
	A	783.48^b^	2.40^ab^	215.77^b^	0.28^b^	0.18^b^	0.70^ab^	88.17^b^	0.0031^b^	0.40^a^	35.78^ab^	26.81^ab^
	B	937.34^a^	2.48^b^	256.98^a^	0.38^a^	0.22^a^	0.77^a^	118.75^a^	0.0025^c^	0.41^a^	37.68^a^	28.35^a^
	Low rainfall	**700.39^b^**	**2.52^a^**	**218.82^b^**	**0.29^b^**	**0.15^b^**	**0.71^a^**	**114.84^a^**	**0.0018^b^**	**0.28^b^**	**35.20^b^**	**25.67^b^**
	High rainfall	**860.41^a^**	**2.44^b^**	**236.37^a^**	**0.33^a^**	**0.20^a^**	**0.73^a^**	**103.46^b^**	**0.0028^a^**	**0.40^a^**	**36.73^a^**	**27.58^a^**

		**LA_Exp.3_**	**TDM**	**SLA**	**Tillers**	**Height**	**Morning TR (10 am)**	**Afternoon TR (3 pm)**	**LA_Exp.4_**			
	**Zone**	**(cm^2^)**	**(g)**	**(cm^2^.g^-1^)**		**(mm)**	**(g H_2_O.mm^-2^)**	**(g H_2_O.mm^-2^)**	**(cm^2^)**			

Outdoors	A1	6536^a^	28.37^a^	230^ab^	7.80^a^	288.86^a^	0.19^a^	0.68^a^	3544^a^			
	A	5752^a^	27.44^a^	246^a^	7.14^a^	266.57^b^	0.18^a^	0.65^a^	3044^b^			
	B	6313^a^	27.80^a^	226^b^	5.45^b^	288.11^ab^	0.20^a^	0.69^a^	2572^c^			
	Low rainfall	**6536^a^**	**28.37^a^**	**230^a^**	**7.80^a^**	**288.86^a^**	**0.19^a^**	**0.68^a^**	**3544^a^**			
	High rainfall	**6541^a^**	**27.62^b^**	**236^a^**	**6.30^b^**	**277.34^a^**	**0.20^a^**	**0.67^a^**	**2808^b^**			

*Comparisons were performed with LSD test (*p* < 0.05), the different superscript letters indicate the means separation. *LA* is the leaf area, TDM is the aerial dry matter, *SLA* is the specific leaf area which is the LA per gram of leaf dry matter and indicates how thin are the leaves. Both these measurements were taken at the beginning of the drydown, on individual plants. *Root shoot* is the ratio between root and aerial dry weight, *Ex* is the xylem exudate, *RDW* is the root dry weight, *RL* is the root length, *Ex-RL* is the xylem exudate normalized by root length, *Ex-RDW* is the xylem exudate normalized by root dry weight, *DM control* is the aerial dry matter under optimal conditions, *DM stress* is the aerial dry matter under water stress due to progressive soil drying, *LA_*Exp.3*_* is the LA in cm equivalent to LA_3d (recorded by the scanner) in Exp.3, *TDM* is the total dry matter. Both *LA_*Exp.3*_* and TDM were measured after the VPD response experiment outdoors on plants of 35 days and recorded for four plants per experimental unit. *Tillers* is the number of tillers developed each the plant, *Height* is the plant height, *Morning TR* is the transpiration rate at 10 am accounting the LA-3d at low VPD conditions, *Afternoon TR* is the transpiration rate at 10 am accounting the LA-3d at high VPD conditions, and *LA_*Exp.4*_* is the LA in cm equivalent to LA_3d in Exp.4.*

**FIGURE 4:**
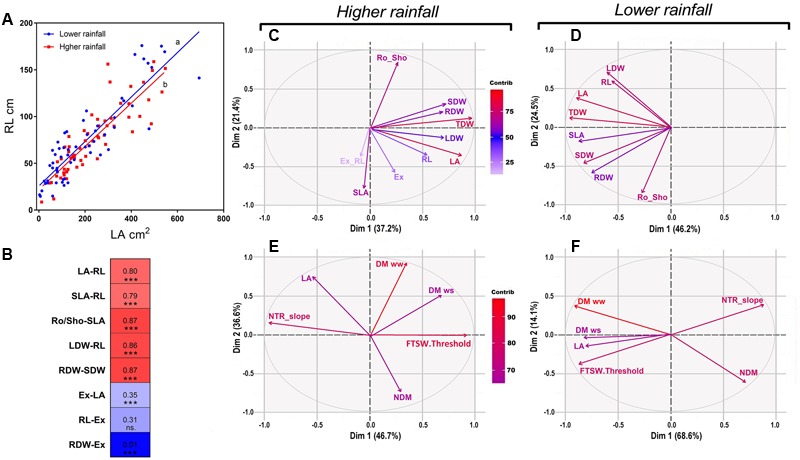
Pearson correlations and PCA analysis of physiological and transpiration related traits under optimal conditions as in the response soil drying between higher and lower rainfall genotypes. **(A)** Shows the coordinated growth leaf-root representing the linear regression between leaf area and root length (RL) within higher rainfall and lower rainfall genotypes, as **(B)** shows the main highest and lowest Pearson’s correlations between phenotypic traits in both zones in a correlation chart between physiological traits. **(C,D)** Explain the influence of physiological traits in Exp.1 for both rainfall zones under optimal conditions; as in water stress the relation of transpiration traits and biomass are shown in **(E,F)**. VPD, vapor pressure deficit; NTR slope, slope of normalized transpiration in the response to soil drying; DM ww, dry matter in optimal conditions; DM ws, dry matter under water stress; NDM, normalized dry matter; FTSW, fraction of transpirable soil water; FSTW threshold, FTSW at which the transpiration of plants exposed to water stress began to decline; LA, leaf area; LDW, leaf dry weight; SDW, stem dry weight; TDM, aerial dry matter; SLA, specific leaf area; Ex, xylem exudation; Ex-RL, exudation normalized by root length; RL, root length; RDW, root dry weight; Ro_Sho, root shoot index; Dim, dimension; contrib, contribution; ww, well-watered; ws, water stress.

According to the Pearson correlations we found a strong significant correlation (0.880; *p* < 0.000) between LA and RL (**Figure 4A**), and more generally strong significant correlations between shoot and root traits (**Figure 4B**). By contrast, poor correlations were found between the net exudation rate with RL (0.3; *p* < 0.001) or with LA (0.35; *p* < 0.000), and no correlation with the RDW (0.01; *p* < 0.855) (**Figure 4B**).

### Physiological Parameters of the Combinations (F1 Hybrid and Parental) from Higher and Lower Rainfall Zones

The physiology of the combinations (F_1_ hybrid and parental) (Supplementary Table S2) showed significant differences between the F_1_ hybrids, B- and R-lines in both rainfall zones (**Table 7**). The F_1_ hybrids showed significant larger values for LA, TDM, RDW, exudate, RL and normalized exudation (Ex-RL, Ex-SDW, Ex-RDW), also while evaluated in response to soil drying under stress and optimal conditions their dry matter was higher than the B- and R-lines, which confirmed the hybrid heterotic effects. In both cases the hybrid showed lower root/shoot ratio than the parental. There was also a large difference between the R lines compared to B and F1 in both zones. Especially in A1 zone, the R-line were the smallest in most of the traits such as LA, TDW, RDW, SLA, exudate and normalized exudation, also under progressive soil drying showed the lowest LA.

**Table 7:** Physiological parameters comparisons between combinations of F1 hybrids and parental (B- and R-lines) that evolved in lower rainfall zone (A1) and higher rainfall zones (A and B).

	Response to high VPD											Response to soil drying			
		LA	TDM	RDW	Root	Exudate	SLA	RL	Ex-RL	Ex-SDW	Ex-RDW	LA	DM stress	DM control	NDM
	Class	(cm^2^)	(g)	(g)	Shoot	(g_._ h^-1^)	(cm^2^.g^-1^)	(cm)	(g.h^-1^.cm^-1^)	(g.h^-1^.g^-1^)	(g.h^-1^.g^-1^)	(cm^2^)	(g)	(g)	
Lower rainfall	F_1_	366.85^a^	2.44^a^	0.45^a^	0.19^c^	0.18^a^	187.40^a^	114.84^a^	0.0018^a^	0.38^a^	0.41^a^	1453.64^a^	25.67^a^	35.20^a^	0.74^b^
	B line	168.19^b^	1.56^b^	0.31^b^	0.20^b^	0.08^b^	127.90^b^	61.10^b^	0.0014^b^	0.27^b^	0.26^b^	1365.73^ab^	22.91^b^	25.13^b^	0.94^a^
	R line	73.13^c^	1.25^c^	0.30^c^	0.24^a^	0.07^c^	68.36^c^	45.93^c^	0.0015^b^	0.25^c^	0.23^c^	834.50^b^	19.03^b^	20.94^b^	0.92^a^
Higher rainfall	F_1_	341.87^a^	2.22^a^	0.41^a^	0.19^c^	0.26^a^	184.36^a^	103.46^a^	0.0028^a^	0.61^a^	0.66^a^	1925.87^a^	27.58^a^	36.73^a^	0.75^b^
	B line	155.38^c^	1.44^c^	0.31^c^	0.23^a^	0.10^b^	126.38^c^	53.92^c^	0.0018^c^	0.36^c^	0.37^c^	2040.01^a^	21.18^b^	27.34^b^	0.80^a^
	R line	211.12^b^	1.75^b^	0.33^b^	0.20^b^	0.14^c^	147.25^b^	71.84^b^	0.0021^b^	0.43^b^	0.53^b^	1635.37^a^	19.56^b^	25.93^b^	0.77^b^

*Left Section shows the parameters of TR response to High VPD (left) and to progressive soil drying (right). Comparisons performed with LSD test (*p* < 0.05), the different superscript letters indicate the means separation. For abbreviation see legend of **Table 6**. NDM is the DM stress normalized by DM control.*

### Comparative Trait Analysis between Higher and Lower Rainfall Zones

A multivariate PCA analysis performed with data of Exp.1 showed the variation of the physiological parameters and their contribution in each rainfall zone. Under well-watered conditions all plant traits had positive loading on the main vector, both for the low and high rainfall hybrids (**Figures 4C,D**). On the second main vector, the different plant traits were distributed across the *X*-axis, with no major difference between low and high rainfall hybrids, except that SLA and the exudation rate had a strong negative loading for the high rainfall hybrids (**Figure 4C**) whereas it had no weight in the low rainfall hybrids (**Figure 4D**). The aerial dry matter (TDW) and LA were the most influent traits in both zones, and RDW was highly influent in low rainfall zone. Under progressive soil drying (**Figures 4E,F**), aerial dry matter (DM), NTR slope and FTSW threshold were the most influent traits in both zones. In low rainfall zone FTSW threshold and LA were located in the same quadrant respect to the main two main vectors (82%) showing their close relation on the main two vectors, while in high rainfall zone these traits were opposite on the main vector (46%) showing their independence.

Moreover, a set of highly significant correlations (see Supplementary Table S1 and **Figure 4B**) between growth traits from Exp.1 and Exp.2 showed coordinated relationships between aerial and root growth. This was also shown in the linear regression between LA and RL (*r*: 0.8^∗∗∗^) represented in **Figure 4A** in both rainfall zones of evolution.

## Discussion

**FIGURE 5:**
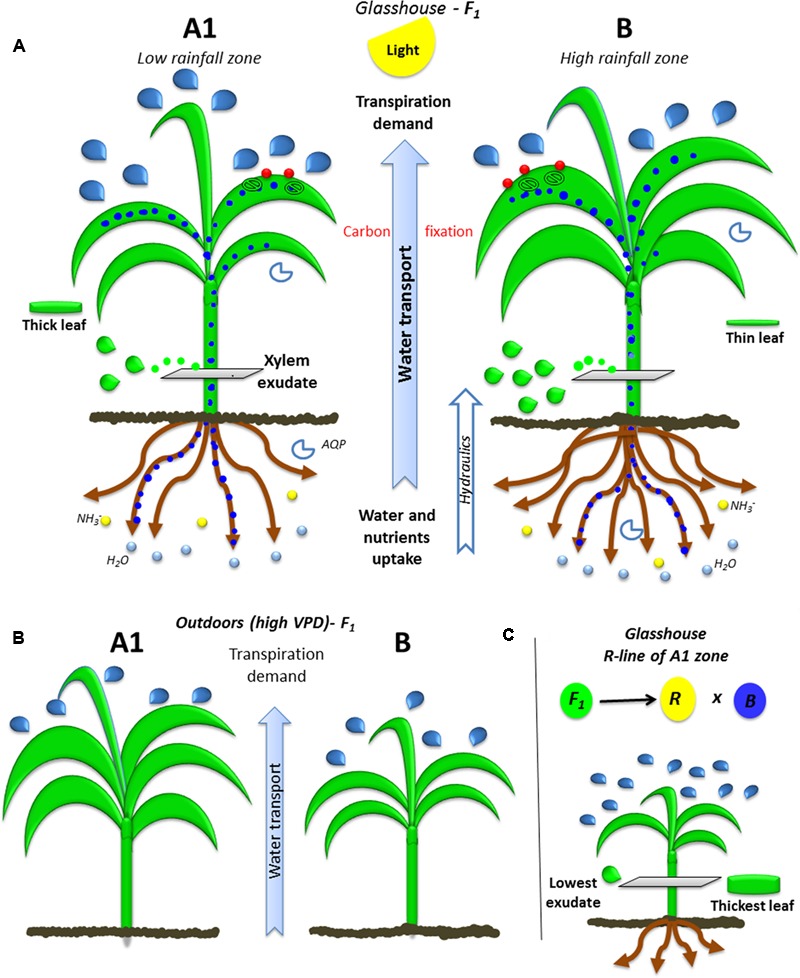
Ideotypes of A1 and B plants bred in high and low rainfall zone. The **(A)** illustrates the water transport (blue dots) from roots to leaves trough xylem (green drops) due to transpiration demand (blue drops: transpiration) and the integration of carbon fixation in the leaves (CO_2_: red points) by the stomata and nutrient uptake by roots (blue circles: water and yellow circles: nitrate) in glasshouse conditions. **(B)** Shows the growth and transpiration ideotypes of A1 and B zone hybrids grown outdoors, **(C)** shows the parental R-line of A1 zone which is the most contrasting line.

A schematized physical and function representation of high and low rainfall hybrids is shown in **Figure 5** as a mean of summarizing the main findings. In brief, the hybrids bred in high (HR) and low rainfall (LR) zones had different transpiration response to high VPD depending on the VPD level of their growth environment (**Figure 5A**), the largest differences were found between the hybrids bred in A1 (LR) and B (HR) zones. When they grew in greenhouse (low VPD), the lower rainfall hybrids transpired more than higher rainfall ones under high VPD conditions, they had smaller LA and biomass, and thinner leaves (higher SLA). Their canopy development under high VPD outdoors was opposite, where lower rainfall hybrids had larger and thicker leaves (LA, TDM, and SLA) than the high rainfall zone hybrids. In addition, the roots (RDW) and xylem exudates (Exudate, Ex_RL and Ex_RDW) were higher in high rainfall hybrids. Regardless of their target breeding zones, genotypes showed a close relationship between root and canopy area, suggesting a coordinated growth between root and shoot. The parental lines were different from the hybrids in most of the traits evaluated, which reflected the heterotic effect, although in the A1 zone parent/hybrid combinations, the R-line was particularly contrasting with F_1_ (**Figure 5B**).

### Transpiration Response to Increases in VPD

When grown in glasshouse conditions, the low rain fall zone hybrids did not restrict the transpiration under increasing VPD conditions whereas the high rainfall zone (B) hybrids did, although the two groups of genotypes did not display any transpiration rate differences under low VPD conditions. By contrast, when the plants were grown in outdoors conditions, the hybrids from the different zones did not display any difference in the transpiration response to increasing VPD. The former observation is consistent with earlier report on a pearl millet hybrid developed for the A1 zone, HHB 67, and which did not display any transpiration restriction under increasing VPD conditions, compared to another line bred for better endowed environment and which displayed a transpiration restriction under high VPD conditions (Kholová et al., 2010a). The interpretation could be made that genetic material having evolved, or being bred, for A1-types of environments where the rainfalls are erratic and in very sandy soil, would have likely developed adaptation strategies favoring a rapid water uptake before the water is lost to either infiltration or soil evaporation. On the contrary, genetic material bred for B-type environments with wetter conditions and deeper soil with higher clay content could have favored a transpiration restriction under high VPD, i.e., when the water cost of fixing carbon is the highest (Vadez et al., 2013). A similar observation could be done from recent report on *Phaseolus* species, where drought adapted lima and tepary beans showed almost no sensitivity to increasing VPD conditions (Medina et al., 2017).

The fact that in outdoors conditions, exposed to hotter/dryer conditions, there was no difference in the transpiration response to increasing VPD conditions between the hybrids developed for different rainfall zones, suggests an interaction between the transpiration response and the VPD conditions prevailing in the growing environment. Our interpretation is that the transpiration demand under high VPD conditions during growth would have prompted the plant development to cater for such a high water demand. Several earlier report can be interpreted in the same way. In a work on turfgrass, Sermons et al. (2012) showed that while plants restricted transpiration under high VPD conditions when grown under cool conditions, close to those of the adaptation zone of that particular specie, the transpiration restriction was much weaker when the plants were grown under higher temperature. Similar observation linking the degree of transpiration control under increasing VPD conditions to the temperature in the growing environment was made in soybean (Seversike et al., 2013). In another study on pearl millet, it was also shown that a number of plant traits were altered by growing in a higher VPD environment, in particular there was less of a transpiration restriction in lines that usually restrict transpiration under increasing VPD, and there was also some effect on the root anatomy (endodermal cell size), which was hypothesized to relate to root hydraulic conductivity differences (Kholova et al., 2016). This need of limiting the stomata closure to maintain higher photosynthetic rate and increase the leaf duration in drought-deciduous species was previously reported in nutrient deficit habitats (Rodriguez-Iturbe et al., 2001). Therefore, more work would be needed to test side by side if there is indeed an effect of the VPD in the growth conditions on the transpiration response to transient step increases in VPD.

### The Absence of Difference in the FTSW Thresholds

Equally important under soil moisture-limited conditions, this water conservative behavior with early stomata closure was the same in both rainfall hybrids; both declined at high soil moisture content and slowly, and a higher penalty on biomass production occurred in lower rainfall genotypes. Previous studies in superior genotypes of pearl millet reported that the lower daily transpiration which consequently drives NTR under drought conditions resulted in lower FTSW thresholds (Kholová et al., 2010a). On the contrary, the fitness of our hybrids showed higher FTSW thresholds and subsequent lower NTR slope upon further decrease in soil moisture. It is not clear why no difference in the FTSW threshold were found. The intuitive hypothesis that genetic material adapted to erratic rainfall pattern, or hybrids bred for the A1 zone here, would favor a behavior of using soil water instead of losing it through soil evaporation, was here rejected. This decline on TR at high soil water content in hybrids was also reported as a phenomenon related to low hydraulic conductance (Choudhary and Sinclair, 2014).

### Growth Strategies

Leaf area and root area were closely related, suggesting that leaf and root growth worked in a closely coordinated manner to respond in both directions to the leaf demand of photosynthesis and transpiration, as the root demand for water and nutrient uptake (**Figure 5A**). This coordinated metabolism was supported in other studies as linked to the leaf stomatal closure, which gathers a signal of abscisic acid (ABA) in the xylem while the roots is sensing the decrease in soil moisture (Laffray and Louguet, 1990). Also according to previous studies in several plant communities (Chenopodiaceae, Poaceae, Fabaceae, and Asteraceae) and plant types (C3 and C4 grasses, and legumes) of Chinese arid and semi-arid zones, plants show a pattern of positive correlation between root and leaf traits like SLA-SRL and nitrogen content in both organs, they also affirmed that the correspondence aboveground– belowground leads to a strong whole-plant economic strategy of conserving or acquiring carbon and nutrient resources (Liu et al., 2010). Recent work in pearl millet also showed that growth under high VPD conditions affected some traits of the root anatomy like the size of the endodermal cells, suggesting indeed a tight linkage in the development of root and shoot traits (Kholova et al., 2016).

Outdoors, exposed to hotter conditions where VPD raised to ∼5 kPa, the lower rainfall hybrids produced more tillers, accumulated more biomass, and had a higher leaf growth (**Figure 5A**). The tillering production is indeed a strategy for successful adaptation to unfavorable environment, where additional reproductive tillers can compensate the loss of panicles to water stress. Earlier report mentions this as a strategy to minimize crop failure that also carries a yield penalty (Van Oosterom et al., 2003; Kim et al., 2010). This strategy is not only under genetic control but also under environmental control in cereals such as sorghum (Van Oosterom et al., 2003; Kim et al., 2010). Contrary results were found under the lower VPD growth conditions of the glasshouse, where high rainfall hybrids developed larger canopy than the low rainfall hybrids. This was consistent with earlier report of lower rainfall hybrids reporting a smaller canopy when grown outdoors during the rainy season in the LeasyScan platform (Vadez et al., 2015). Our interpretation is similar to the one above to explain the absence of transpiration restriction under high VPD in the low rainfall hybrids: In A1-types of environments, with likely frequent events of high VPD conditions (between rain gaps), adapted genotypes are likely to be those that are able to sustain leaf expansion. It is known that high VPD conditions restrict leaf expansion in maize, although there is large genotypic variation (Reymond et al., 2003; Caldeira et al., 2014). Then under low VPD conditions, it is also understandable that high rainfall hybrid would be those developing a larger canopy to maximize light capture in an environment that does not have water limitation. This would also be supported by the higher exudate rate of these high rainfall hybrids in the low VPD growth conditions. This is where also the size could explain in part the differences in the transpiration response to increasing VPD between the low and high rainfall hybrids, where larger canopy B-hybrids would have a propensity to have restricted transpiration under high VPD because of canopy size. The difference in the transpiration response to increasing VPD between the hybrids and their parents, and the fact that R-lines showed much higher transpiration rates slope response to increasing VPD than their hybrids, would comfort this interpretation.

### Differences in the Combinations of Hybrids and Parental Lines

Our experiments showed different behavior in vegetative stage between the parental and the hybrids, where the hybrids showed their heterotic superiority in biomass production. Interestingly, our study suggests that the ability to regulate the TR could have been conferred by any of the two parental lines, in our lower rainfall genotypes this capacity is given by the B line parent. Contrasting with the higher rainfall genotypes where the donor is the restorer parent. So the high slope of response in the A1 hybrid could be driven by the R line? During the last decades, some studies conducted in the development history of hybrids reported that the line x pollinator interaction was not significant for grain either biomass under favorable conditions, and there was low heritability in stress scenarios in the north part of India, which is our low rainfall zone (Yadav et al., 2000). Later the single cross based on *CMS (cytoplasmic male sterility)* technique was improved to *single top crosses* with top restorer lines, because mostly the breeding programs looked for yield increment in non-extreme environments. However, the approach in the Indian breeding of these hybrids was the adaptation to arid zone via restorer line (R line) which are arid zone landraces and confer the adaptive characters (increased productivity) to the hybrid (Yadav et al., 2009) this is the cross type of the population used in this experiment.

## Conclusion

In this investigation we have shown that the breeding history had an impact on several traits playing a central role in plant water use. High rainfall hybrids did restrict transpiration under high evaporative demand while low rainfall hybrids did not, and the latter were also able to maintain a larger canopy under high evaporative demand. These traits in the low rainfall hybrids could also be interpreted as part of a strategy of adaptation to low and erratic rainfall consisting of maximizing water use when available. Such results open an opportunity to include such traits as part of the breeding selection process.

## Author Contributions

SM and VV conceived and designed the experiment; SKG provided the breeding material, pedigree data and data of production environments. SM conducted the experimental work. SM and VV contributed to the data analysis and interpreted the results. SM wrote the paper under the supervision of VV. All authors revised the manuscript, read and approved the final manuscript.

## Conflict of Interest Statement

The authors declare that the research was conducted in the absence of any commercial or financial relationships that could be construed as a potential conflict of interest.

## Footnotes

1 www.graphpad.com
