# Supplementary material for: Transpiration Response and Growth in Pearl Millet Parental Lines and Hybrids Bred for Contrasting Rainfall Environments
Source: Front Plant Sci. 2017 Oct 30;8:1846. doi: 10.3389/fpls.2017.01846 (PMC5671031; doi:10.3389/fpls.2017.01846)

Transpiration response and growth under varying vapour pressure deficit in pearl millet parental lines and hybrids bred for contrasting rainfall environments

Susan Medina[1,2], S K Gupta[1], Vincent Vadez[1*].

[1]International Crops Research Institute for semi-arid Tropics (ICRISAT), Crop Physiology Laboratory, Patancheru 502324, Greater Hyderabad, Telangana, India.

[2]Integrative Crop Ecophysiology Group, Plant Physiology Section, Faculty of Biology, University of Barcelona, Barcelona, Spain.

*Corresponding author:
Vincent Vadez
International Crops Research Institute for semi-arid Tropics (ICRISAT), Crop Physiology Laboratory, Patancheru 502324, Greater Hyderabad, Telangana, india.
E-mail: V.VADEZ@cgiar.org
Type of article: Original Research Article

Number of figures: 5 (5 in color)
Figure 1 (1.5 columns)
Figure 2 (single column)
Figure 3 (1.5 columns)
Figure 4 (2 columns)
Figure 5 (1.5 columns)

Number of tables: 7 (0 in color)
Supplementary material: 1 figure and 3 tables
Figure 1S (2 column)

Supplementary Table 1 Correlation Analysis by Pearson Method – Two sided of genotypes (parental and F1 hybrid) evolved in higher rainfall zone (A1) and lower rainfall zones (A and B) during 2014 and 2015.

Correlation matrix		LA	SLA	LDW	RDW	RL	Ro_Sho	SDW	TDW	Ex	Ex-LA	Ex-LDW	Ex-RDW	Ex-RL	Ex-SDW	Ex-TDW
Cor	**LA**	1	0.310	0.800	0.460	**0.880**	-0.030	0.510	0.850	0.350	-0.440	0.090	0.050	0.040	-0.020	-0.030
p value		1	0.000	0.000	0.000	**0.000**	0.631	0.000	0.000	0.000	0.000	0.188	0.461	0.693	0.800	0.663
Cor	**SLA**	0.310	1	-0.090	0.230	0.790	**-0.870**	0.180	0.080	0.150	-0.220	**0.850**	-0.010	0.130	-0.360	**-0.900**
p value		0.000	1	0.207	0.001	0.000	**0.000**	0.010	0.269	0.025	0.002	**0.000**	0.913	0.154	0.000	**0.000**
Cor	**LDW**	0.800	-0.090	1	0.110	0.860	0.110	0.110	0.690	0.290	-0.250	-0.180	0.180	0.010	0.210	0.190
p value		0.000	0.207	1	0.104	0.000	0.116	0.097	0.000	0.000	0.000	0.009	0.011	0.910	0.002	0.006
Cor	**RDW**	0.460	0.230	0.110	1	0.760	0.180	**0.870**	0.710	**0.010**	-0.450	-0.010	-0.350	-0.070	-0.350	-0.020
p value		0.000	0.001	0.104	1	0.000	0.008	**0.000**	0.000	**0.855**	0.000	0.898	0.000	0.464	0.000	0.737
Cor	**RL**	0.880	0.790	0.860	0.760	1	-0.310	0.750	0.870	**0.310**	-0.370	0.090	0.130	-0.070	0.100	0.100
p value		0.000	0.000	0.000	0.000	1	0.001	0.000	0.000	**0.001**	0.000	0.306	0.173	0.457	0.258	0.266
Cor	**Ro_Sho**	-0.030	-0.870	0.110	0.180	-0.310	1	0.240	0.240	-0.090	-0.040	-0.840	-0.110	-0.090	0.240	0.950
p value		0.631	0.000	0.116	0.008	0.001	1	0.000	0.000	0.200	0.556	0.000	0.105	0.306	0.000	0.000
Cor	**SDW**	0.510	0.180	0.110	0.870	0.750	0.240	1	0.800	0.040	-0.430	-0.060	-0.310	-0.060	-0.360	0.080
p value		0.000	0.010	0.097	0.000	0.000	0.000	1	0.000	0.559	0.000	0.389	0.000	0.501	0.000	0.268
Cor	**TDW**	0.850	0.080	0.690	0.710	0.870	0.240	0.800	1	0.210	-0.470	-0.150	-0.120	-0.010	-0.140	0.170
p value		0.000	0.269	0.000	0.000	0.000	0.000	0.000	1	0.003	0.000	0.028	0.081	0.927	0.050	0.014
Cor	**Ex**	0.350	0.150	0.290	0.010	0.310	-0.090	0.040	0.210	1	0.210	0.510	0.800	0.860	0.720	0.120
p value		0.000	0.025	0.000	0.855	0.001	0.200	0.559	0.003	1	0.002	0.000	0.000	0.000	0.000	0.080
Cor	**Ex_LA**	-0.440	-0.220	-0.250	-0.450	-0.370	-0.040	-0.430	-0.470	0.210	1	0.140	0.500	0.440	0.440	0.110
p value		0.000	0.002	0.000	0.000	0.000	0.556	0.000	0.000	0.002	1	0.041	0.000	0.000	0.000	0.129
Cor	**Ex_LDW**	0.090	0.850	-0.180	-0.010	0.090	-0.840	-0.060	-0.150	0.510	0.140	1	0.400	0.950	0.060	-0.740
p value		0.188	0.000	0.009	0.898	0.306	0.000	0.389	0.028	0.000	0.041	1	0.000	0.000	0.364	0.000
Cor	**Ex_RDW**	0.050	-0.010	0.180	-0.350	0.130	-0.110	-0.310	-0.120	0.800	0.500	0.400	1	0.860	**0.860**	0.150
p value		0.461	0.913	0.011	0.000	0.173	0.105	0.000	0.081	0.000	0.000	0.000	1	0.000	0.000	0.032
Cor	**Ex_RL**	0.040	0.130	0.010	-0.070	-0.070	-0.090	-0.060	-0.010	0.860	0.440	0.950	0.860	1	0.890	0.950
p value		0.693	0.154	0.910	0.464	0.457	0.306	0.501	0.927	0.000	0.000	0.000	0.000	1	0.000	0.000
Cor	**Ex_SDW**	-0.020	-0.360	0.210	-0.350	0.100	0.240	-0.360	-0.140	0.720	0.440	0.060	0.860	0.890	1	0.490
p value		0.800	0.000	0.002	0.000	0.258	0.000	0.000	0.050	0.000	0.000	0.364	0.000	0.000	1	0.000
Cor	**Ex_TDW**	-0.030	-0.900	0.190	-0.020	0.100	0.950	0.080	0.170	0.120	0.110	-0.740	0.150	0.950	0.490	1
p value		0.663	0.000	0.006	0.737	0.266	0.000	0.268	0.014	0.080	0.129	0.000	0.032	0.000	0.000	1

Cor: Pearson correlation coefficient
p value: significance

Supplementary Table 2 Physiological parameters of genotype triplets (parental and F1hybrid) evolved in higher rainfall zone (A1) and lower rainfall zones (A and B) during 2014.

ID	LA (cm^2)	TDW (g)	RDW (g)	Root Shoot	Ex (g. h^{-1})	SLA ($cm^2.g^{-1}$)	RL (cm)	Ex-RL (g .h^{-1}.cm^{-1})	Ex-SDW (g.h^{-1}.g^{-1})	Ex-RDW (g .h^{-1}.g^{-1})
1	381.880 ± 9.240	2.534 ± 0.046	0.438 ± 0.005	0.179 ± 0.003	0.283 ± 0.011	193.366 ± 1.707	108.440 ± 2.903	0.00298 ± 0.00012	0.544 ± 0.022	0.672 ± 0.026
2	183.108 ± 7.071	1.700 ± 0.040	0.334 ± 0.004	0.211 ± 0.004	0.080 ± 0.004	126.318 ± 1.861	77.381 ± 1.053	0.00103 ± 0.00006	0.272 ± 0.017	0.242 ± 0.013
3	97.146 ± 3.418	1.402 ± 0.026	0.276 ± 0.007	0.196 ± 0.004	0.072 ± 0.007	86.976 ± 2.747	37.829 ± 0.770	0.00165 ± 0.00012	0.200 ± 0.014	0.246 ± 0.018
4	232.280 ± 8.222	1.656 ± 0.038	0.376 ± 0.008	0.232 ± 0.004	0.203 ± 0.009	166.477 ± 3.281	77.247 ± 2.548	0.00265 ± 0.00017	0.604 ± 0.029	0.480 ± 0.018
5	86.183 ± 1.595	1.110 ± 0.008	0.234 ± 0.006	0.210 ± 0.005	0.051 ± 0.003	94.588 ± 1.589	34.960 ± 1.237	0.00146 ± 0.00006	0.246 ± 0.011	0.222 ± 0.008
6	24.121 ± 1.656	0.884 ± 0.009	0.244 ± 0.007	0.284 ± 0.008	0.043 ± 0.002	31.472 ± 2.002	25.558 ± 0.943	0.00149 ± 0.00005	0.252 ± 0.013	0.169 ± 0.006
7	475.389 ± 8.746	3.016 ± 0.049	0.512 ± 0.008	0.172 ± 0.003	0.149 ± 0.011	210.478 ± 1.569	152.411 ± 1.757	0.00094 ± 0.00007	0.217 ± 0.016	0.300 ± 0.021
8	173.640 ± 4.114	1.634 ± 0.030	0.328 ± 0.009	0.211 ± 0.006	0.054 ± 0.005	134.938 ± 1.510	58.832 ± 1.521	0.00079 ± 0.00006	0.142 ± 0.011	0.145 ± 0.010
9	71.022 ± 2.564	1.284 ± 0.016	0.318 ± 0.005	0.248 ± 0.003	0.067 ± 0.005	66.342 ± 1.753	57.837 ± 1.534	0.00125 ± 0.00009	0.259 ± 0.019	0.227 ± 0.017
10	377.858 ± 12.429	2.560 ± 0.053	0.482 ± 0.012	0.189 ± 0.002	0.070 ± 0.003	179.266 ± 3.847	121.253 ± 4.174	0.00078 ± 0.00005	0.145 ± 0.007	0.174 ± 0.009
11	229.843 ± 6.549	1.808 ± 0.027	0.332 ± 0.004	0.189 ± 0.003	0.147 ± 0.003	155.759 ± 3.062	73.250 ± 1.466	0.00225 ± 0.00009	0.420 ± 0.014	0.451 ± 0.010
12	100.249 ± 4.234	1.418 ± 0.029	0.346 ± 0.005	0.254 ± 0.004	0.102 ± 0.005	88.669 ± 2.015	62.484 ± 1.862	0.00149 ± 0.00006	0.295 ± 0.013	0.286 ± 0.014
13	309.957 ± 12.043	2.068 ± 0.057	0.338 ± 0.013	0.157 ± 0.003	0.094 ± 0.006	178.573 ± 2.439	85.369 ± 3.402	0.00147 ± 0.00011	0.244 ± 0.015	0.378 ± 0.029
14	246.546 ± 8.018	1.832 ± 0.034	0.308 ± 0.005	0.177 ± 0.004	0.050 ± 0.003	168.657 ± 2.226	75.508 ± 1.786	0.00069 ± 0.00004	0.139 ± 0.010	0.153 ± 0.007
15	222.336 ± 6.917	1.870 ± 0.025	0.328 ± 0.008	0.179 ± 0.005	0.100 ± 0.003	140.207 ± 2.528	71.626 ± 1.507	0.00150 ± 0.00007	0.300 ± 0.008	0.354 ± 0.017
16	324.482 ± 11.517	2.186 ± 0.058	0.368 ± 0.004	0.184 ± 0.004	0.363 ± 0.016	180.995 ± 3.209	90.964 ± 2.976	0.00468 ± 0.00021	0.823 ± 0.039	0.951 ± 0.034
17	60.745 ± 2.863	1.056 ± 0.019	0.260 ± 0.002	0.261 ± 0.005	0.057 ± 0.005	68.007 ± 2.691	24.424 ± 1.063	0.00190 ± 0.00010	0.231 ± 0.015	0.220 ± 0.018
18	220.570 ± 7.356	1.878 ± 0.023	0.346 ± 0.009	0.182 ± 0.004	0.164 ± 0.012	147.740 ± 2.883	78.610 ± 1.604	0.00236 ± 0.00021	0.467 ± 0.038	0.715 ± 0.075
19	407.960 ± 7.315	2.366 ± 0.034	0.474 ± 0.007	0.202 ± 0.002	0.312 ± 0.019	206.149 ± 1.290	121.435 ± 2.381	0.00292 ± 0.00019	0.838 ± 0.050	0.722 ± 0.043
20	158.432 ± 5.556	1.272 ± 0.027	0.298 ± 0.007	0.250 ± 0.006	0.188 ± 0.015	151.178 ± 1.388	55.344 ± 1.858	0.00303 ± 0.00017	0.771 ± 0.059	0.864 ± 0.089
21	198.648 ± 7.069	1.496 ± 0.040	0.334 ± 0.010	0.227 ± 0.003	0.096 ± 0.004	159.092 ± 1.848	64.916 ± 2.317	0.00149 ± 0.00002	0.347 ± 0.008	0.291 ± 0.008
22	325.077 ± 10.559	2.276 ± 0.049	0.452 ± 0.010	0.204 ± 0.003	0.261 ± 0.013	171.740 ± 2.592	116.072 ± 2.640	0.00217 ± 0.00009	0.559 ± 0.022	0.600 ± 0.031
23	155.793 ± 5.364	1.608 ± 0.030	0.360 ± 0.005	0.229 ± 0.002	0.100 ± 0.003	117.680 ± 1.684	60.420 ± 1.537	0.00170 ± 0.00006	0.288 ± 0.005	0.271 ± 0.007
24	202.937 ± 5.127	1.752 ± 0.021	0.324 ± 0.004	0.191 ± 0.004	0.213 ± 0.010	141.955 ± 1.740	72.228 ± 1.675	0.00290 ± 0.00014	0.589 ± 0.030	0.754 ± 0.047

2014 Hybrids $F_{1\ (1,4,7,10,13,16,19,22)}$ – B line $_{(2,5,8,11,14,17,20,23)}$ R line $_{(3,6,9,12,15,18,21,24)}$

ID	Genotypes	ID	Genotype	ID	Genotype
**Zone A1**		*9*	*88004 B*	*17*	*ICMB 04222*
1	*HOPE 2013-AHT-R-8*	*10*	*HHB 67 imp*	*18*	*JBV 3 S1 -237-1-3-3-1-B*
2	*96666 B*	*11*	*843-22 B*	**Zone B**	
3	*RIB 3135/18*	*12*	*H77/833-2-202*	*19*	*AHT-II/K13-5*
4	*HOPE-2013 AHT-R-14*	**Zone A**		*20*	*ICMB 99222*
5	*843-22 B*	*13*	*AHT A/K13-4*	*21*	*ICMV 96490-S1-15-1-2-1-1*
6	*MRC S1-97-3-4-B-B-1-B-1-B*	*14*	*ICMB 97222*	*22*	*AHT-II/K13-24*
7	*HOPE-2013 AHT-R-18*	*15*	*MRC HS-130-2-2-1-B-B-3-B-B-B-1-3-1*	*23*	*ICMB 98222*
8	*(EERC-HS-29)-B-13-4-5-2*	*16*	*AHT A/K13-5*	*24*	*(MC 94 C2-S1-3-2-2-2-1-3-B-B x AIMP 92901 S1-488-2-1-1-4-B-B)-B-2-2-2*

Supplementary Table 3 Physiological parameters of F1- Hybrids evolved in higher rainfall zone (A1) and lower rainfall zones (A and B) during 2015.

ID	LA (cm^2)	TDW (g)	SDW (g)	RDW (g)	Root Shoot	Ex ($g.h^{-1}$)	SLA ($cm^2.g^{-1}$)	Ex-SDW ($g\text{-}h^{-1}.g^{-1}$)	Ex-RDW ($g.h^{-1}.g^{-1}$)
**101**	324.248 ± 6.750	2.766 ± 0.056	1.540 ± 0.028	0.989 ± 0.014	0.364 ± 0.002	0.141 ± 0.004	271.648 ± 3.419	0.098 ± 0.004	0.151 ± 0.006
**102**	298.372 ± 6.934	2.602 ± 0.050	1.433 ± 0.018	1.036 ± 0.020	0.426 ± 0.012	0.130 ± 0.004	260.001 ± 2.240	0.093 ± 0.003	0.135 ± 0.005
**103**	258.952 ± 4.600	2.299 ± 0.026	1.283 ± 0.009	0.751 ± 0.007	0.328 ± 0.001	0.149 ± 0.006	255.563 ± 1.109	0.112 ± 0.004	0.188 ± 0.007
**104**	257.448 ± 2.408	2.497 ± 0.027	1.378 ± 0.014	0.984 ± 0.012	0.395 ± 0.003	0.111 ± 0.003	233.252 ± 1.617	0.084 ± 0.003	0.117 ± 0.004
**105**	246.598 ± 4.042	2.552 ± 0.028	1.545 ± 0.030	0.921 ± 0.015	0.370 ± 0.007	0.153 ± 0.005	252.771 ± 5.907	0.105 ± 0.004	0.177 ± 0.006
**106**	298.520 ± 6.018	2.877 ± 0.024	1.552 ± 0.020	1.180 ± 0.019	0.409 ± 0.005	0.125 ± 0.006	228.198 ± 2.941	0.085 ± 0.004	0.117 ± 0.006
**107**	306.356 ± 10.047	2.669 ± 0.033	1.397 ± 0.006	1.042 ± 0.005	0.398 ± 0.004	0.094 ± 0.006	237.753 ± 3.402	0.068 ± 0.004	0.095 ± 0.006
**108**	308.218 ± 5.461	2.716 ± 0.017	1.376 ± 0.005	1.047 ± 0.009	0.386 ± 0.003	0.101 ± 0.004	230.292 ± 3.057	0.074 ± 0.003	0.099 ± 0.004
**109**	334.934 ± 7.030	2.702 ± 0.034	1.441 ± 0.012	1.089 ± 0.013	0.410 ± 0.005	0.123 ± 0.007	266.028 ± 3.067	0.084 ± 0.004	0.122 ± 0.008
**110**	290.312 ± 4.104	2.299 ± 0.017	1.099 ± 0.029	0.912 ± 0.016	0.408 ± 0.010	0.069 ± 0.002	245.145 ± 4.109	0.066 ± 0.002	0.080 ± 0.003
**111**	340.242 ± 5.704	2.856 ± 0.057	1.533 ± 0.030	1.137 ± 0.034	0.391 ± 0.006	0.166 ± 0.003	267.025 ± 3.582	0.115 ± 0.003	0.171 ± 0.006
**112**	340.824 ± 5.247	2.798 ± 0.050	1.492 ± 0.028	1.030 ± 0.019	0.367 ± 0.001	0.200 ± 0.005	264.301 ± 2.405	0.145 ± 0.006	0.212 ± 0.008
**113**	280.062 ± 0.759	2.454 ± 0.022	1.362 ± 0.005	1.165 ± 0.007	0.479 ± 0.004	0.079 ± 0.002	266.409 ± 3.750	0.059 ± 0.001	0.068 ± 0.002
**114**	340.850 ± 4.809	2.087 ± 0.087	0.988 ± 0.046	1.014 ± 0.027	1.339 ± 0.273	0.160 ± 0.006	747.338 ± 75.653	0.180 ± 0.044	0.198 ± 0.012
**115**	247.846 ± 2.658	2.643 ± 0.026	1.384 ± 0.009	0.951 ± 0.035	0.355 ± 0.011	0.127 ± 0.002	212.083 ± 5.730	0.093 ± 0.002	0.166 ± 0.007
**116**	341.684 ± 4.159	2.914 ± 0.031	1.511 ± 0.020	1.075 ± 0.016	0.371 ± 0.005	0.233 ± 0.009	245.137 ± 1.847	0.162 ± 0.007	0.237 ± 0.012
**117**	341.040 ± 9.892	2.959 ± 0.075	1.538 ± 0.038	1.021 ± 0.028	0.345 ± 0.004	0.179 ± 0.007	239.397 ± 2.543	0.116 ± 0.003	0.185 ± 0.006
**118**	289.562 ± 6.172	2.731 ± 0.051	1.459 ± 0.021	1.266 ± 0.044	0.460 ± 0.011	0.124 ± 0.001	239.718 ± 4.089	0.089 ± 0.002	0.114 ± 0.003

2015 Hybrids (F_1)

ID Zone A1	Genotypes	ID Zone A	Genotype	ID Zone B	Genotype
1	HOPE-2014 AHT-R-15	9	AHT II/K14-7	17	IHT B1 /K14-20
2	HOPE-2014 AHT-R-7	10	AHT A/K14-5	18	AHT II/K14-20
3	HOPE-2014 AHT-R-11	11	AHT II/K14-9	19	IHT B1 /K14-10
4	HOPE 2013-AHT-R-8	12	IHT A2 /K14-24	20	AHT-II/K13-5
5	HOPE-2013 AHT-R-14	13	AHT A/K13-4	21	ICMH 1201
6	HHB 67 imp	14	AHT A/K13-5	22	AHT-II/K13-24

Supplemental figures legend.

Supplementary **Figure 1. Individual profile of soil drying response of each genotype evolved in low and high rainfall zones of India.** The upper panels show the dry down response of combinations [F_1 hybrids (blue), B-line or sterile male (red) and R-line or restorer (green)] bred in higher rainfall zone, and the bottom panels show the ones bred in lower rainfall zones. Each biological replicate is shown as a circle and it segmented regression is shown as a line. NTR: normalized transpiration rate, FTSW fraction of transpirable soil water.

TR transpiration rate, FTSW fraction of transpirable soil water

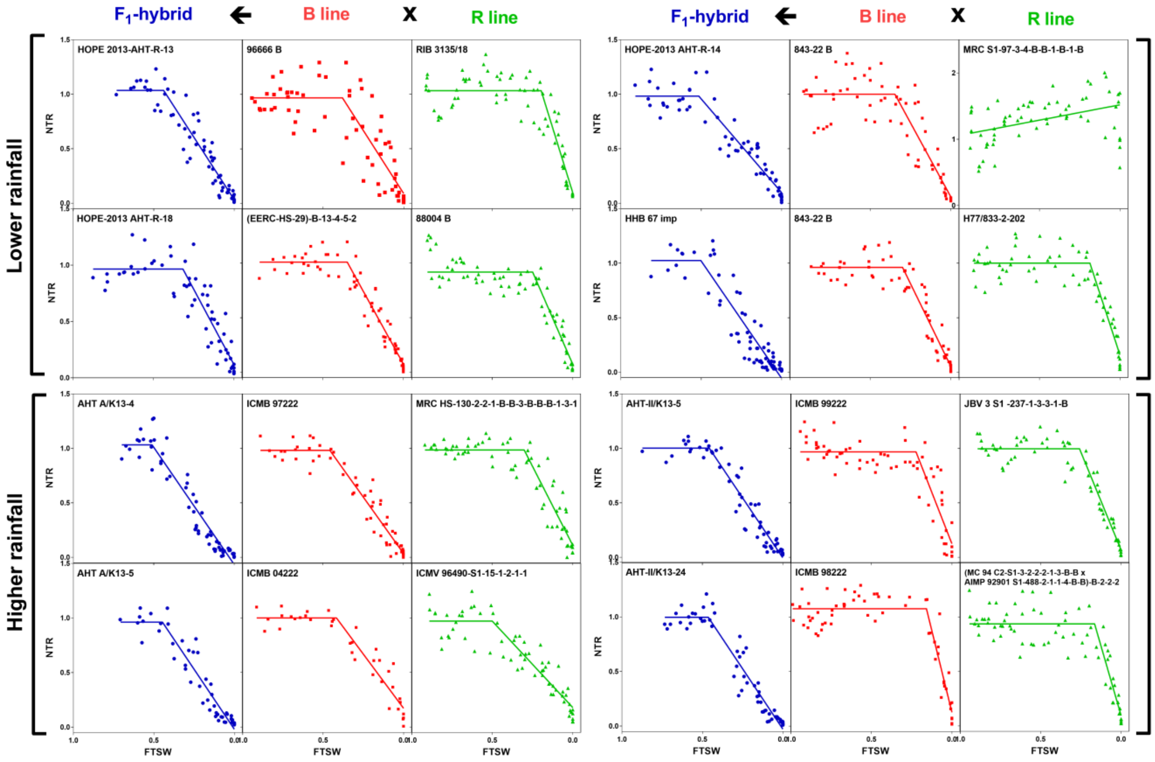

Supplement: Supplementary file 1 [file Supplementary_Material.PDF]
